# Intrinsically Nonswellable Multifunctional Hydrogel with Dynamic Nanoconfinement Networks for Robust Tissue‐Adaptable Bioelectronics

**DOI:** 10.1002/advs.202207237

**Published:** 2023-02-17

**Authors:** Jae Park, Ju Yeon Kim, Jeong Hyun Heo, Yeonju Kim, Soo A Kim, Kijun Park, Yeontaek Lee, Yoonhee Jin, Su Ryon Shin, Dae Woo Kim, Jungmok Seo

**Affiliations:** ^1^ School of Electrical and Electronic Engineering Yonsei University Seoul 03722 Republic of Korea; ^2^ LYNK Solutec inc. Seoul 03722 Republic of Korea; ^3^ Department of Chemical and Biomolecular Engineering Yonsei University Seoul 03722 Republic of Korea; ^4^ Department of Physiology Yonsei University College of Medicine Seoul 03722 Republic of Korea; ^5^ Division of Engineering in Medicine Department of Medicine Brigham and Women's Hospital Harvard Medical School 65 Lansdowne Street Cambridge MA 02139 USA

**Keywords:** 3D printing, bioelectronics, hydrogels, self‐healing, tissue adhesives

## Abstract

Developing bioelectronics that retains their long‐term functionalities in the human body during daily activities is a current critical issue. To accomplish this, robust tissue adaptability and biointerfacing of bioelectronics should be achieved. Hydrogels have emerged as promising materials for bioelectronics that can softly adapt to and interface with tissues. However, hydrogels lack toughness, requisite electrical properties, and fabrication methodologies. Additionally, the water‐swellable property of hydrogels weakens their mechanical properties. In this work, an intrinsically nonswellable multifunctional hydrogel exhibiting tissue‐like moduli ranging from 10 to 100 kPa, toughness (400–873 J m^−3^), stretchability (≈1000% strain), and rapid self‐healing ability (within 5 min), is developed. The incorporation of carboxyl‐ and hydroxyl‐functionalized carbon nanotubes (fCNTs) ensures high conductivity of the hydrogel (≈40 S m^−1^), which can be maintained and recovered even after stretching or rupture. After a simple chemical modification, the hydrogel shows tissue‐adhesive properties (≈50 kPa) against the target tissues. Moreover, the hydrogel can be 3D printed with a high resolution (≈100 µm) through heat treatment owing to its shear‐thinning capacity, endowing it with fabrication versatility. The hydrogel is successfully applied to underwater electromyography (EMG) detection and ex vivo bladder expansion monitoring, demonstrating its potential for practical bioelectronics.

## Graphical abstract

This study reports a nonswellable multifunctional hydrogel for bioelectronics. The hydrogel is soft yet tough and self‐healable, so it can softly and robustly interface with tissues. Its conductivity and tissue adhesiveness further ensure precise biosignal detection. Additionally, the hydrogel is three‐dimensional (3D) printable, ensuring its versatile fabricability. It demonstrates reliable real‐time sensing of organ volume and electrophysiological signals.

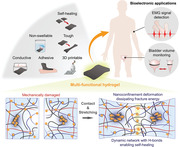

## 1 Introduction

Recent remarkable advances in bioelectronic devices have facilitated the growth of the healthcare industry.^[^ ^1^ ^]^ Accordingly, demands for practical bioelectronics enabling daily healthcare monitoring have surged. These so‐called smart healthcare devices require long‐term functionality within the human body.^[^ ^2^ ^]^ However, existing bioelectronics lack tissue adaptability owing to intrinsically different natures of electronic materials and biological tissues.^[^ ^3^ ^]^ For example, robustly interfacing planar‐shaped stiff electronics and curvilinear soft tissue surfaces under dynamic tissue environments is arduous.^[^ ^4^ ^]^ Moreover, the abundant water in the tissue environment further hinders strong interfacing with dry electronics, and the water can degrade the performance of the bioelectronics. Bridging these disparities and enhancing robust tissue adaptability should be achieved to ensure the long‐term functionality of bioelectronics.

In this regard, soft electronics (e.g., elastomers and conductive polymers) have been widely investigated to softly interface bioelectronics with the human body.^[^ ^5^ ^]^ Nevertheless, limitations exist in achieving complete biointerfacing between bioelectronics and tissues because even bioelectronics considered “soft” show a higher order of magnitude of Young's moduli (approximately 1 MPa–1 GPa) than those of biological tissues (≈100 kPa).^[^ ^6^ ^]^ Hydrogels have recently emerged as promising next‐generation soft bioelectronics owing to their extremely soft mechanical properties in the modulus range of kilopascals and water‐rich nature.^[^ ^7^ ^]^ The tissue‐like softness of hydrogels can conformally fit and be stably interfaced with various soft‐tissue surfaces. However, conventional hydrogels possess several limitations in bioelectronics applications in terms of mechanical durability and electrical conductivity. For example, water‐rich hydrogels are usually brittle and weak and thus cannot endure dynamic tissue environments.^[^ ^8^ ^]^ In addition, although ions can be incorporated into the water in hydrogels, reliable electrical conductivity cannot be guaranteed compared to conventional metallic bioelectronic materials.^[^ ^9^ ^]^ Moreover, hydrogels easily swell in wet tissue environments, thereby weakening their mechanical properties and intended functionalities.^[^ ^10^ ^]^ Importantly, various existing well‐established electronic‐fabrication methods are not compatible with hydrogels; therefore, a huge hurdle exists in the precise integration of hydrogels into bioelectronics.^[^ ^6^ ^]^

Recently, various functional hydrogels have been developed to overcome the aforementioned limitations. For example, tough hydrogels with enhanced mechanical durability have recently emerged.^[^ ^8^, ^11^ ^]^ They can endure external forces frequently exerted in the dynamic tissue environments through additional sacrificial bonds. When the hydrogels are irreversibly damaged or ruptured in harsh tissue environments, they can no longer serve bioelectronic functionalities. To address this issue, self‐healing hydrogels with dynamic networks composed of reversible bonds have been designed to restore their initial functionalities after the damage.^[^ ^12^ ^]^ Further, to endow hydrogels with reliable conductivity, the incorporation of conductive nanomaterials (e.g., carbon nanotubes (CNTs), graphene, and metal nanoparticles) into hydrogels has been investigated.^[^ ^13^ ^]^ Despite numerous studies reporting advanced functional hydrogels, accomplishing all the superior functionalities simultaneously is challenging. There is a trade‐off between toughness and self‐healing because a toughening strategy introducing additional chain networks can hinder dynamic chain movements, which are required for self‐healing.^[^ ^14^ ^]^ Incorporating conductive nanomaterials into hydrogels can also restrict the dynamic network due to their weak interactions with the polymer matrix.^[^ ^15^ ^]^ In addition, these nanomaterials compromise the stretchability and softness of hydrogels, although their mechanical strength is increased.^[^ ^16^ ^]^ Even if all the trade‐offs are addressed, water swelling of the hydrogel can deteriorate its optimized multifunctionality in vivo. Importantly, hydrogels are usually in bulk form, and their precise fabrication methodologies are not well established.^[^ ^6^ ^]^ Thus, adopting hydrogels in the industrial field is challenging.

In this work, we developed an all‐in‐one, intrinsically nonswellable hydrogel with 3D printability for long‐term‐functioning practical bioelectronics. The hydrogel comprises hydrophilic polymers such as poly(vinyl alcohol) (PVA) and poly(acrylic acid) (PAA), tannic acid (TA), and functionalized carbon nanotube (fCNT). fCNT is highly functionalized CNT with abundant carboxyl and hydroxyl groups via oxidation with KMnO_4_.^[^ ^17^ ^]^ TA and fCNT can be distributed within the overall hydrogel network by interacting through hydrogen bonds (H‐bonds) owing to their rich carboxyl or hydroxyl groups. Notably, hydrophobic benzene moieties of TA and fCNT promote nanophase separation against hydrophilic polymers, and hydrophobic interactions and *π*‐stackings further result in high‐density TA and fCNT aggregates forming nanoconfinement structures. The nanoconfinement structures provide effective energy dissipation for the toughening property of the hydrogel (400–873 J m^−3^). In the nonconfined region, abundant hydroxyl groups of each component interact reversibly via H‐bond interaction, thereby enabling rapid self‐healing (within 5 min). Considering that the existing rapidly self‐healing hydrogels generally have high flowability and are vulnerable to plastic deformation, the hydrogel possessing toughness and fast self‐healing ability holds a high potential for its practical applications. Moreover, well‐dispersed fCNTs over the entire network ensure high electrical conductivity (≈40 S m^−1^). With the chemical functionalization of the polymer chain, strong adhesiveness (≈50 kPa) of the hydrogel to various device materials and tissue surfaces can be achieved, which reinforces further robust interfacing with tissues. Furthermore, hydrophobic moieties in the TAs and fCNTs allow the hydrogel to be swelling‐resistant, thus enabling underwater self‐healing and improving durability in wet tissues. The hydrogel shows a shear‐thinning property above 50 °C for 3D printing with micron precision owing to the cleavage of abundant H‐bonds within the network. By combining these desirable functionalities, the hydrogel was reliably applied to bioelectronic applications including ex vivo bladder volume detection and electromyography (EMG) signal sensing. The hydrogel strongly interfaced with the target tissues owing to its robust tissue adaptability and provided clear signals. Considering the high‐resolution 3D printability of the hydrogel, its multifunctionality can be easily incorporated into the complex forms of sophisticated bioelectronics for future smart healthcare systems.

## 2 Results and Discussion

### 2.1 Multifunctional Hydrogel Design and Characterization

**Figure 1:**
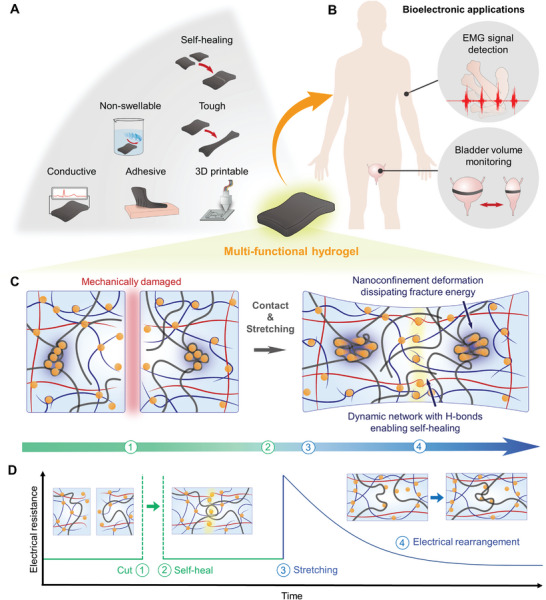
Overall concept of the hydrogel. A) The multifunctionality of the hydrogel. B) Bioelectronic applications of the hydrogel for EMG signal detection and bladder volume monitoring. C) Schematic illustration of the dynamic nanoconfinement network of the hydrogel. The ruptured hydrogel autonomously self‐heals and shows high toughness owing to the unique network: 1) mechanically damaged, 2) self‐heals upon contacting the cut hydrogels, 3) stretching the self‐healed hydrogel, and 4) the hydrogel endures a large deformation as the nanoconfinement structure dissipates the fracture energy. D) Schematic illustration of the hydrogel recovering its electrical conductivity under cutting and stretching.

**Figure** **1**A shows the schematic illustration of the innately nonswellable multifunctional hydrogel. The nonswellability, self‐healability, and toughness of the hydrogel ensure its mechanical durability for enduring harsh and wet in vivo conditions for long durations. By combining the softness of the hydrogel with its adhesiveness and conductivity, it can be conformably and robustly interfaced with biological tissues and precisely record biosignals. Figure 1B illustrates various potential bioelectronic applications of the developed hydrogel. Because of its strong tissue adaptability, the hydrogel can be stably interfaced with soft tissues, thus enabling versatile biosignal detection such as physical (e.g., strain and pressure) and electrophysiological (e.g., electroencephalogram (EEG), electrocardiogram (ECG), and EMG). Generally, designing durable soft materials has been considered challenging owing to several trade‐offs across softness and mechanical strength.^[^ ^18^ ^]^ In addition, existing self‐healing hydrogels exhibit a liquid‐like nature because flowability ensures chain movements for self‐healing of the hydrogel network.^[^ ^19^ ^]^ Thus, accomplishing both tough and self‐healing hydrogels is also contradictory. However, the developed hydrogel achieves all properties and can provide practical bioelectronic applicability. This is attributed to the unique dynamic nanoconfinement network of the hydrogel (Figure 1C). The hydrogel can self‐heal from mechanical damage to completely restore its initial state owing to its dynamic network that comprises abundant reversible crosslinkers. Notably, the hydrogel exhibits high toughness during stretching even after self‐healing. There exist locally aggregated nanoconfinement structures based on weak yet dense reversible bonds within the hydrogel network. Nanoconfinement structures can also be found in natural materials such as silk fiber, which overcomes the trade‐off between softness and toughness.^[^ ^20^ ^]^ The silk fiber consists of nanoconfined *β*‐sheet crystals embedded in a semiamorphous matrix. Although the silk fiber network mainly relies on weak H‐bonds, the highly dense nanoconfinement structure endows high toughness to the silk fiber by effectively and reversibly dissipating high fracture energies.^[^ ^21^ ^]^ Similarly, as the external forces are applied to the hydrogel, breakage, and reformation of highly dense nanoconfinement structures repetitively dissipate fracture energies, allowing large deformation while maintaining high elasticity. Interestingly, as these nanoconfinements are locally aggregated, the entire dynamic networks are conserved and the hydrogel can exhibit rapid self‐healing and toughness. Furthermore, we noticed that the hydrogel not only mechanically self‐healed, but also reconstructed its electrical properties (Figure 1D). Generally, mechanical damage and stretching of the hydrogel can lead to breakage of the conductive pathway.^[^ ^22^ ^]^ However, the hydrogel restores its electrical pathways during self‐healing and stretching. As the fCNT can dynamically interact within the network via H‐bonds, its conducting pathway can be rapidly reconstructed. This suggests that the hydrogel possesses electrical stability as well as mechanical durability for long‐term functioning bioelectronics.

**Figure 2:**
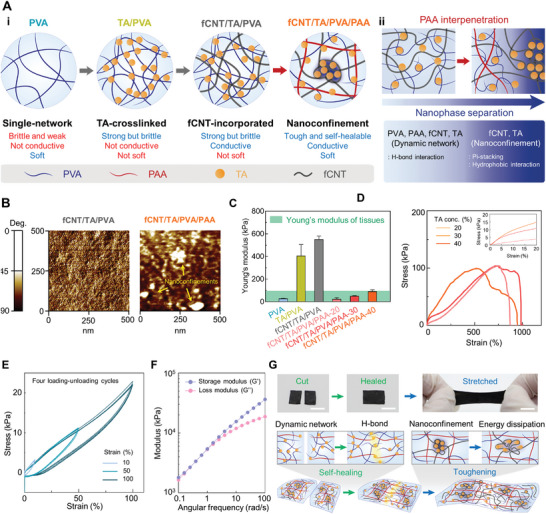
Fabrication of the hydrogel and its characterization. A‐i) Schematic illustration describing fabrication steps of the hydrogel. A‐ii) Schematic illustrating nanophase separation upon interpenetration of PAA into fCNT/TA/PVA. B) Representative AFM phase image mode images of fCNT/TA/PVA and fCNT/TA/PVA/PAA. C) Young's moduli of hydrogels in each fabrication step (n = 3: n is the sample size for each group). D) Tensile test measurement of the developed hydrogel with varying TA concentrations. The inset shows a magnified graph for the strain of ≈20%. E) Four cycles of cyclic tensile–compressive tests at 10%, 50%, and 100% strains. G) Rheological analysis of moduli as a function of frequency. F) Photographs and schematic of the hydrogel under cutting, self‐healing, and stretching (scale bar: 5 mm).

**Figure** **2**A(i) shows a schematic illustration of the hydrogel fabrication process. PVA hydrogels capable of forming H‐bonding networks have been widely studied owing to their tissue‐like softness and biocompatibility.^[^ ^23^ ^]^ However, PVA hydrogels are mechanically weak; accordingly, various strategies to improve their mechanical properties have been reported.^[^ ^24^ ^]^ We added TA as a reversible and high‐density H‐bonding crosslinker into the PVA hydrogel network. TA is a catechol and pyrogallol‐rich natural compound having 25 hydroxyl groups in a single molecule, thus forming dynamic networks based on abundant reversible H‐bonds within the hydrogel.^[^ ^13^, ^25^ ^]^ A TA/PVA hydrogel with rich H‐bond crosslinks exhibits enhanced mechanical strength. Next, we introduced fCNT to endow electrical conductivity to the hydrogel. fCNT can be well dispersed in water up to 50 mg mL^−1^ owing to its rich carboxyl and hydroxyl groups. This enables fCNT to be uniformly distributed within the hydrogel to provide a sufficiently conductive pathway. However, the high‐density crosslinking of TA and mechanically strong nature of fCNT increases Young's moduli of the TA/PVA and fCNT/TA/PVA hydrogels, which exceed that of soft tissues (10–100 kPa). After additionally interpenetrating a PAA polymer, hydrophilic–hydrophobic nanophase separation occurs because of local aggregations of fCNT and TA through *π*‐stacking and hydrophobic interaction (Figure 2A(ii)). The aggregations form a nanoconfinement network within the fCNT/TA/PVA/PAA hydrogel; thus, it is mechanically tough, soft, and self‐healable. The nanoconfinement structures of the hydrogel were observed using atomic force microscopy (AFM) in the phase image mode. Figure 2B shows the AFM phase image mode of the fCNT/TA/PVA and fCNT/TA/PVA/PAA. The darker region with a low phase angle represents relatively soft parts, and the brighter region with a high phase angle reveals stiff parts. In accordance with our assumption, while the fCNT/TA/PVA showed a uniformly distributed network, distinct nanophase separation was verified after the interpenetration of PAA into the fCNT/TA/PVA. Transmission electron microscopy (TEM) was additionally used to visualize the nanomorphology of the fCNT/TA/PVA/PAA hydrogel and confirmed the nanoconfinement structures (Figure S1, Supporting Information). The high‐magnification image shows uniform lattice structures, indicating the overall dispersion of fCNT. This implies that the fCNT is distributed within the entire hydrogel network while locally concentrated nanoconfinement exists. To investigate the chemical bonding in the hydrogel, Fourier transform infrared (FT‐IR) spectroscopy and X‐ray photoelectron spectroscopy (XPS) was performed (Figures S2 and S3, Supporting Information). After the introduction of TA into the PVA hydrogel, FT‐IR showed weakening and broadening of the stretching vibration peak at 3319 cm^−1^, indicating increased H‐bonds of hydroxyl groups between PVA and TA.^[^ ^26^ ^]^ In addition, phenolic O—H and C=O bending peaks were observed after introducing TA and fCNT, which have phenols and carbonyl groups. This was also confirmed by XPS showing the appearance of the C=O peak (≈288.5 eV) after the introduction of TA and fCNT. After interpenetration of the PAA chain into the fCNT/TA/PVA hydrogel, the intensity of the C—C peak (≈284.8 eV) was found to be higher than that of the C—O peak (≈286 eV), implying that the PAA polymer chains were well introduced. We hypothesized that the nanophase separation would ensure tissue‐like softness of the fCNT/TA/PVA/PAA compared to the densely crosslinked TA/PVA and fCNT/TA/PVA. Accordingly, we measured Young's moduli of the hydrogels in each step of fabrication to validate the hypothesis (Figure 2C). The soft PVA hydrogel had higher Young's moduli after the introduction of TA and fCNT. Although the mechanical strengths of the hydrogels improved, their Young's moduli exceeded that of soft tissues and they could not precisely form an interface with the tissues. Interestingly, further interpenetration of PAA into fCNT/TA/PVA resulted in a decrease in Young's modulus in the range of that of soft tissues. Further, we noted that by varying the concentration of the TA crosslinkers (e.g., fCNT/TA/PVA/PAA‐20 denotes 20% w/v TA), the moduli of the fCNT/TA/PVA/PAA hydrogel could be modulated within the range of Young's moduli of soft tissues (10–100 kPa). This modulus tunability enables conformal interfacing with a wide range of various soft tissues, which are not mechanically matched with conventional electronic materials (Figure S4, Supporting Information). Figure 2D shows the results of the mechanical tensile test demonstrating the tissue‐like Young's moduli and toughness of the hydrogel. The inset in the graph shows its tunable modulus according to varying TA concentrations. The hydrogel exhibited a high tensile strength of approximately 95–121 kPa with an elongation strain of ≈1000%. Based on the rubber elasticity theory, increasing the polymer chain density increases Young's modulus.^[^ ^27^ ^]^ Therefore, the conventional approach of introducing additional chain networks for toughening inevitably leads to a higher Young's modulus. Nevertheless, the developed hydrogel overcomes the trade‐off and has both toughness and softness. This is attributed to the locally aggregated nanoconfinement network dissipating fracture energy, while the relatively low density of the nonconfined region ensures the soft modulus of the hydrogel. We noticed that the density and size of nanoconfinement structures cannot be precisely controlled for each sample because of the abundant H‐bonds and coagulating nature of TA. Thus, dissipating properties and the tendency of enduring large strains could slightly vary. Still, Young's modulus and stress behavior at initial strains (≈30%) are reproducible according to crosslinking density of TA (Figure S5, Supporting Information). We further investigated the toughness of the hydrogel through cyclic tensile–compressive loading tests for 10%, 50%, and 100% strains (Figure 2E). The results of the test showed its repeatable dissipation in that dissipated energies calculated by an inner area of the curve were almost maintained during the multiple cycles. During the cycles, the hydrogel showed a hysteresis of only ≈2.5%. The deformation of the energy‐dissipating nanoconfinements can be immediately restored because it comprises high‐density reversible H‐bonds. This suggests that the hydrogel can reliably serve as a tough hydrogel and endure in human‐body conditions under the application of frequent loads. As the hydrogel did not undergo plastic deformation until 100% strain, the test also demonstrated a repeatable elastic behavior of the hydrogel. Given that existing soft hydrogels generally lack elasticity and are vulnerable to irreversible deformation, the developed soft yet elastic hydrogel holds remarkable practicality for various biomedical applications. Rheological analysis was further performed to evaluate the elasticity of the hydrogel (Figure 2F). The rheological curves of the angular frequency‐dependent storage modulus (G’) and loss modulus (G″) at 25 °C were measured. In all the frequency regions, G’ was higher than G″, which also confirms elastic solid behavior of the hydrogel.^[^ ^28^ ^]^ The G’ decreases and proximates with G″ at the lower frequency (≈ 1 rad s^−1^), which is attributed to viscoelasticity and stress‐relaxation behavior of the hydrogel.^[^ ^29^ ^]^ The longer probed time with stress application at low frequencies results in dissociation and restructure of the reversibly crosslinked hydrogel network, thus the hydrogel slightly shows viscous behavior at low frequencies.^[^ ^30^ ^]^ This viscoelastic nature corresponds with the soft yet tough properties of the hydrogel with the dynamic network. Additionally, the dynamic nanoconfinement network enables to overcome the trade‐off between toughness and self‐healing because the nonconfined region involves dynamic H‐bond interactions. Figure 2G shows optical images and schematics of the fCNT/TA/PVA/PAA hydrogel showing its self‐healing and toughening abilities. The nonconfined dynamic region allows rapid autonomous self‐healing through reversible H‐bonds, and the nanoconfinement structure effectively dissipates fracture energy during the hydrogel deformation, leading to high toughness. The hydrogel not only has tissue‐like softness but also possesses various unique mechanical properties, suggesting the superior robust tissue adaptability of the hydrogel.

### 2.2 Mechanical Durability

**Figure 3:**
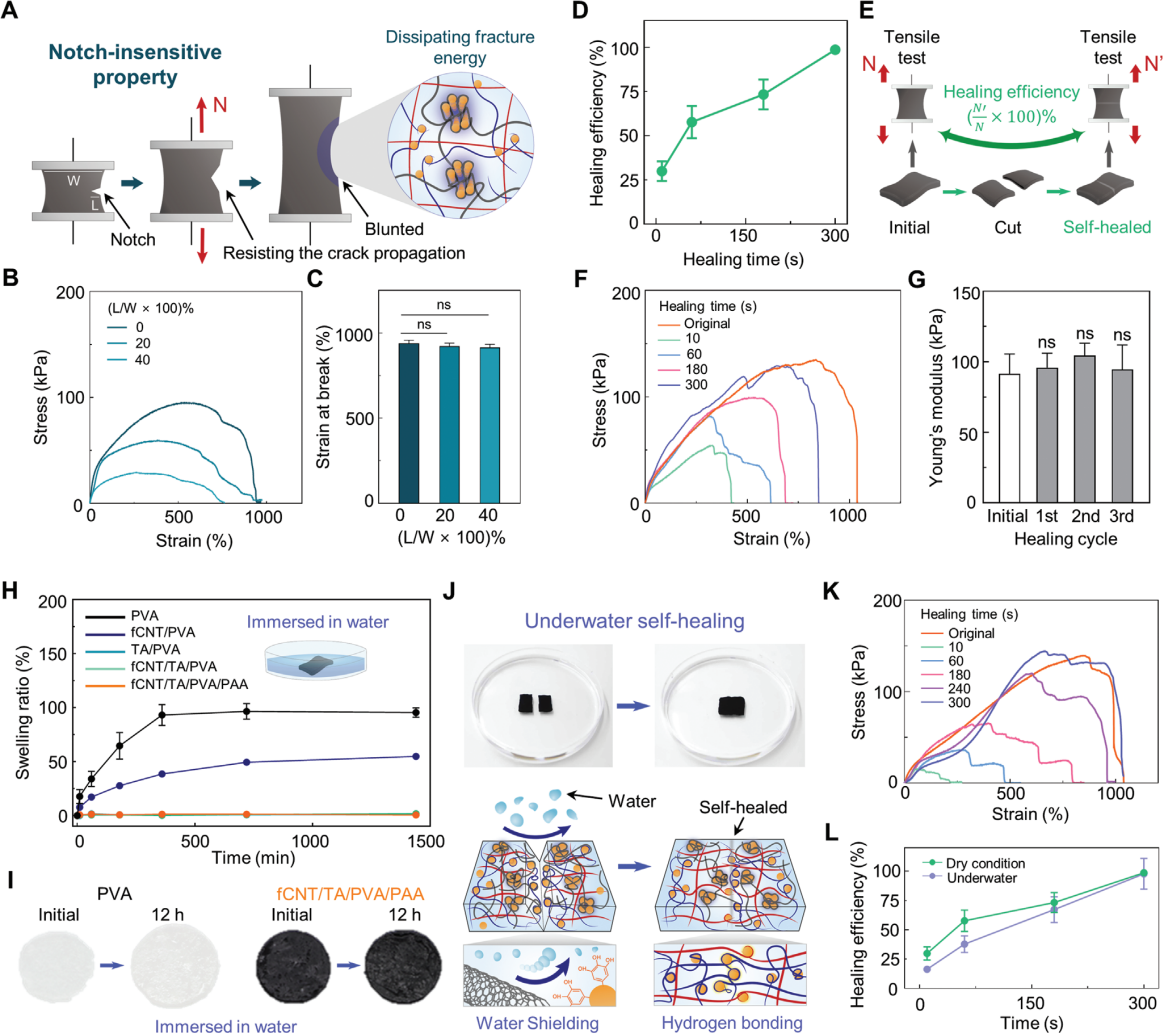
Investigations of mechanical durability of the hydrogel. A) Schematic illustration describing the notch‐insensitive property of the hydrogel. B) Tensile tests of notch‐introduced hydrogels with varying notch lengths. C) Statistical analysis of the strain‐at‐break of the notch‐introduced hydrogels. D) Self‐healing efficiency of the hydrogel as a function of healing time (n = 3: n is the sample size for each time point). E) Schematic illustration of a method for measuring the self‐healing efficiency. F) Tensile test results of initial and self‐healed hydrogel with varying healing times. G) Young's modulus statistics of hydrogels for each healing cycle (n = 3: n is the sample size for each group). H) Swelling ratio of hydrogels with different components (n = 3: n is the sample size for each group and time point). I) Optical image showing swelling behavior of PVA and fCNT/TA/PVA/PAA hydrogels immersed in water for 12 h. J) Photograph and schematic of underwater self‐healing hydrogel. K) Stress–strain curves of underwater self‐healed hydrogels for each healing time. L) Self‐healing efficiency of the hydrogel in dry and underwater conditions as a function of time (n = 3: n is the sample size for each group and time point). Statistical analyses were performed with unpaired t‐tests (*P < 0.05, **P < 0.01, ***P < 0.001, and ****P < 0.0001; ns: no significant difference).

A main hurdle in hydrogels being applied in practical bioelectronics is mechanical durability. Water‐rich hydrogels are usually brittle and weak. The dynamic tissue environments with frequent movements can damage such weak hydrogels and affect their performance. In particular, conventional hydrogels are vulnerable to the formation of a notch, which easily initiates crack propagation, resulting in their mechanical failure.^[^ ^31^ ^]^ This notch‐sensitive property of the hydrogels easily causes their mechanical failure within dynamic tissue environments. Moreover, hydrogels cannot deal with irreversible mechanical failure. Thus, hydrogels have been regarded to have limitations in long‐term bioelectronic applications. In addition, as hydrogels are vulnerable to water swelling, their mechanical properties can be weakened in wet tissue environments.^[^ ^32^ ^]^ The developed hydrogel endures under these dynamic and wet tissue environments owing to its outstanding mechanical durability and intrinsic nonswellability. The developed hydrogel exhibits notch‐insensitive nature owing to its toughening property through effective fracture energy dissipation. We observed that as the hydrogel with a notch was stretched, the notch was blunted and did not propagate further (Figure S6, Supporting Information). When the strain is applied to the hydrogel with a notch, the hydrogel will be deformed and nanoconfinements around the notch will significantly dissipate energy to resist the crack propagation (**Figure** **3**A). We introduced a notch length of 20% and 40% of total width of the hydrogel for the notch‐insensitivity test. Despite the introduction of 20% and 40% length of notches with respect to the total width, the hydrogel maintained its initial strain at break (Figure 3B,C). Only a slight decrease in stress was observed owing to the decreased cross‐sectional area of the hydrogel. The results suggest that the hydrogel can robustly endure in the human body even if a slight damage is introduced. Further, the hydrogel can deal with irreversible mechanical failure because it can rapidly self‐heal to reconstruct its initial mechanical properties as previously mentioned. Next, we verified the self‐healing efficiency of the hydrogel (Figure 3D). The healing efficiency was calculated by comparing the original fracture stress and the fracture stress after self‐healing (Figure 3E). A fractured hydrogel autonomously self‐healed 30% of its initial state upon contact within 10 s and almost perfectly recovered after 5 min. Figure 3F shows the results of the tensile test conducted on the hydrogels after healing times of 10, 60, 180, and 300 s. The hydrogel perfectly restored its initial tissue‐like softness as Young's modulus of the hydrogel was maintained after several cycles of self‐healing (Figure 3G). This demonstrates that the hydrogel completely reconstructs its original structure rather than only connecting mechanically. Generally, self‐healing materials naturally show liquid‐like properties with low viscosity because chain mobility within the materials should be accomplished.^[^ ^19^, ^33^ ^]^ The more solid‐like the materials become, the more the movements of the chain are restricted and impede self‐healing ability. As liquid‐like materials lack mechanical strength and elasticity, practical utilization of self‐healing materials in bioelectronic applications has been challenging. The developed hydrogel achieves significant mechanical strength, elasticity, and self‐healability, which has been considered contradictory owing to the dynamic nanoconfinement network. Another critical issue in soft bioelectronics is dealing with the existence of water in tissue environments. In particular, hydrogels easily swell by uptaking water and their mechanical properties weaken.^[^ ^34^ ^]^ Interestingly, our hydrogel exhibits nonswellable nature due to hydrophobic moieties in fCNT and TA. To verify the water‐shielding effect of the fCNT and TA, we measured the swelling ratio of PVA, TA/PVA, fCNT/PVA, fCNT/TA/PVA, and fCNT/TA/PVA/PAA (Figure 3H). The hydrophilic PVA hydrogel showed a swelling ratio of 100%. The swelling ratio dramatically decreased with the introduction of fCNT and TA. In particular, we noticed that TA dominantly contributed to the water‐shielding effect. fCNT/PVA swelled until the swelling ratio of 60%, while TA/PVA showed almost no increase in its weight. Even after the final interpenetration of PAA, the nonswellability was maintained. Figure 3I and Figure S7, Supporting Information, show the optical images of PVA and the developed hydrogel within 12 h of water immersion. PVA—a representative general hydrogel—expands its volume with an increase in immersion time, while the developed hydrogel maintains its volume. We further investigated the mechanical properties of the hydrogel immersed in water (Figure S8, Supporting Information). The hydrogel retained its tensile strength and Young's modulus under water for 13 days. The nonswellability also addresses the long‐term storage issue of hydrogels, which was an obstacle in hydrogel commercialization. General hydrogels easily dry in air and swell in water; thus, they cannot be stored for long periods. The developed hydrogel can be well stored in water, thus holding potential to be utilized in practical bioelectronics. Though, the hydrogel is vulnerable to water loss in the air as existing general hydrogels. Encapsulating the hydrogel with polydimethylsiloxane (PDMS) can be a solution to address this issue thereby ensuring practical wearable bioelectronics.^[^ ^35^ ^]^ We characterized the mass ratio of the hydrogel and the encapsulated hydrogel as a function of time, and the PDMS extensioned the hydrogel's water retention period (Figure S9, Supporting Information). By using the PDMS‐encapsulated hydrogel as wearable patch, the lifetime of the hydrogel can be prolonged in dry conditions. Conventional self‐healing materials based on H‐bonds are incapable of self‐healing under water owing to the hindrance of water molecules. The nonswellable hydrogel with a water‐shielding effect blocks water molecules to ensure underwater self‐healing.^[^ ^36^ ^]^ Without disturbance of the water molecules, the hydrogel can effectively exhibit its self‐healing ability (Figure 3J). We conducted tensile test to verify the underwater self‐healing efficiency of the hydrogel (Figure 3K,L). At the initial phase of healing time, the healing efficiency slightly decreased compared to that under dry conditions, but the hydrogel revealed complete self‐healing under water within 5 min as under dry conditions. Previously, a self‐healing elastomer based on PDMS with a hydrophobic backbone exhibiting a water‐shielding effect has been reported.^[^ ^37^ ^]^ However, it shows slow self‐healing speed, requiring up to 48 h for complete healing due to its lack of chain mobility and low H‐bonding density. The developed hydrogel exhibits rapid underwater self‐healing and thus can be practically applied to various wet tissues. The overall results suggest that the hydrogel can be robustly applied to dynamic and wet tissue environments and exhibit mechanical durability. This can contribute to long‐term bioelectronic applications, thereby further broadening its applicability even in daily human activities.

### 2.3 Electrical Performances

**Figure 4:**
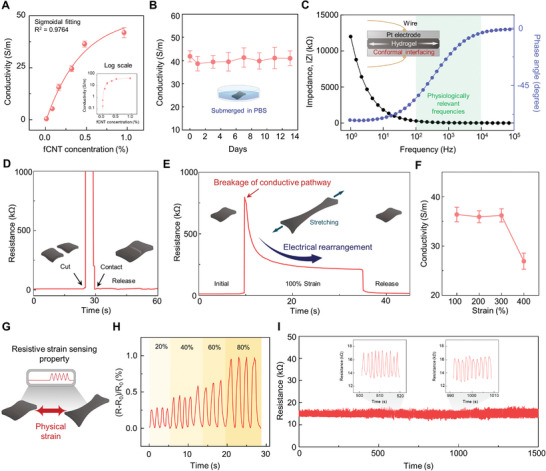
Electrical performances of the hydrogel. A) Quantification of conductivity of the hydrogel as a function of fCNT concentration, fitted with a nonlinear sigmoidal curve (*R* ^2^ = 0.9764; n = 3: n is the sample size for time point). B) Conductivity of the hydrogel submerged in PBS for over 13 days (n = 3: n is the sample size for each point). C) EIS measurement of the hydrogel placed between two platinum electrodes. D) Real‐time resistance measurement demonstrating the hydrogel restoring its electrical property after self‐healing. E) Resistance characterization under stretching of the hydrogel at 100% strain and releasing it. F) Conductivity of the hydrogel at 100%, 200%, 300%, and 400% strains (n = 3: n is the sample size for each point). G) Schematic illustration of the hydrogel as a strain sensor. H) Normalized resistance responses of the hydrogel against 20%, 40%, 60%, and 100% strain. I) Stability of strain‐sensing property of the hydrogel showing repeatable resistive responses over multiple stretchings.

Various electrically conductive hydrogels have been developed for bioelectronics. However, existing hydrogels lack electrical conductivity compared to conventional electronic materials. Furthermore, even if the desired conductivity of hydrogels is achieved, their electrical properties can be lost by mechanical damage caused by dynamic movements of the human body. Thus, high conductivity and its stability should be addressed for designing practical hydrogel bioelectronics. We first visualized the electrical conductivity of the hydrogel by connecting it with a light emitting diode (LED) bulb (Figure S10, Supporting Information). Then, we optimized the conductivity of the hydrogel by modulating the fCNT concentration. **Figure** **4**A shows the conductivity of the hydrogel under the variation of fCNT concentrations with a nonlinear sigmoid fitting parameter of *R* ^2^ = 0.9764. The result revealed a low percolation threshold at 0.25% fCNT concentration, which is attributed to the well‐dispersive nature of the fCNT abundantly functionalized with carboxyl and hydroxyl groups. As the fCNT concentration reached the percolation threshold, the hydrogel exhibited a conductivity of ≈40 S m^−1^. The conductivity was also maintained under phosphate buffer saline (PBS) solution, indicating electrical reliability of the hydrogel under physiological conditions (Figure 4B). The conductivity of tissues is in the range of 0.3–0.7 S m^−1^, and a conductivity over 1 S m^−1^ is known to be sufficient for detecting electrophysiological signals from the human body.^[^ ^38^ ^]^ Thus, the hydrogel possesses suitable electrical properties for bioelectrical signal detections. To further investigate the electrophysiological signal‐detection efficacy of the hydrogel, we conducted electrochemical impedance spectroscopy (EIS) by placing the hydrogel between two platinum electrodes (Figure 4C). Achieving a low impedance at physiologically relevant frequencies (10^2^–10^5^ Hz) is required for bioelectrical signal sensing. The hydrogel with high conductivity can conformally interface with the platinum electrodes, thereby further reducing interconnection and contact resistance. Accordingly, the hydrogel exhibited a low impedance of ≈200 Ω at frequencies of 10^2^–10^5^ Hz. This suggests that the hydrogel does not generate any undesirable interface impedances and can be used for precise bioelectrical signal detection.^[^ ^39^ ^]^ Importantly, we noticed that the hydrogel could robustly maintain its electrical properties even if mechanical deformation and failure were applied to it. As rich H‐bonding functional groups in the fCNT actively interact within the hydrogel network, the dynamic network also allows immediate rearrangement of the conducting pathway of the hydrogel. This was characterized by real‐time resistance measurements. Once the hydrogel was cut into two pieces, the resistance rose sharply owing to the absence of a conducting pathway of fCNTs. Under contact between the two pieces to self‐heal, the resistance immediately recovered to the original value (Figure 4D). The resistance recovery implies that the fCNT pathway is dynamically reconstructed owing to rich H‐bonding groups in the fCNT. Additionally, the dynamic reconstruction of the fCNT pathway was observed when the hydrogel was elongated. Upon stretching the hydrogel to over 100% strain, the resistance of the hydrogel temporally surged because of the breakage of the fCNT pathway. However, within 20 s, the resistance gradually decreased, and after releasing the stretched hydrogel, the resistance recovered to the initial value (Figure 4E). This remarkable electrical reconstruction originates from the dynamic network of the hydrogel combined with the innate characteristics of fCNT. CNTs having a high aspect ratio are known to contribute to a considerably lower percolation threshold compared to other conductive nanomaterials such as metallic nanoparticles because of the increase in probability of overlapping between CNTs.^[^ ^13^, ^40^ ^]^ Accordingly, the disconnected fCNTs can more easily overlap owing to their close proximity and dynamic H‐bonding interactions. The autonomous and rapid reorganization of the conducting pathway within tens of seconds has not been reported previously. Recently, Bao and colleagues have reported PDMS‐based self‐healable CNT composites, which can autonomously reconstruct the disconnected conductive pathway.^[^ ^41^ ^]^ Compared to these previously reported PDMS‐based CNT elastomer composites capable of electrical rearrangement (within 1000 s), the proposed hydrogel exhibits rapid reconstruction (within 20 s) of electrical properties. To the best of our knowledge, our hydrogel is the first material that can rapidly self‐heal and immediately reconnect electrical pathways. We further tested the electrical conductivity of the hydrogel under stretching to more than 100% strain and releasing (Figure 4F). The conductivity measurement was conducted after having enough time for the hydrogel to rearrange the broken conductive pathway. The hydrogel retained its conductivity up to 400% strain. At 400% strain, the conductivity decreased by approximately 10 S m^−1^, indicating that the conducting pathway was irreversibly cleaved over 400% strain. Considering that most tissue deformations are within 100% strain, the hydrogel can maintain its electrical properties during its bioelectronic applications. This was also verified through the real‐time resistance measurement while applying sequential strains of 200%, 300%, and 400% to the hydrogel (Figure S11, Supporting Information). The rearranged resistances after 200% and 300% strains are proximate to the initial resistance, while the resistance after the 400% strain becomes higher. Additionally, electromechanical tests revealed a proportional relationship between the electrical resistance and mechanical stretching of the hydrogel, suggesting that the hydrogel could serve as a strain sensor (Figure 4G). The hydrogel demonstrated a linear relationship between resistance and strain within the threshold strain range of 100%. (Figure S12, Supporting Information). Figure 4H shows the resistive response of the hydrogel for tensile strains of 20%, 40%, 60%, and 100%. The gauge factor of the hydrogel is ≈1.17 which is less than widely adopted intrinsically metallic strain sensors, yet it is comparable to hydrogel‐based strain sensors (≈0.44–1.51).^[^ ^42^ ^]^ Although hydrogel‐based strain sensors have a low gauge factor, they can endure large strains up to 100%, thus can broaden their applicability. The strain sensor property of the hydrogel was further examined to be durable through multiple stretching cycles (Figure 4I). The hydrogel stably maintained its resistive strain sensor property over numerous cycles of stretching owing to its mechanical elasticity. From the electrical performance investigation, we can conclude that the hydrogel could offer reliable and robust electrical properties and also be utilized as a body motion‐detecting strain sensor.

### 2.4 Adhesive and 3D‐Printable Properties of the Hydrogel

**Figure 5:**
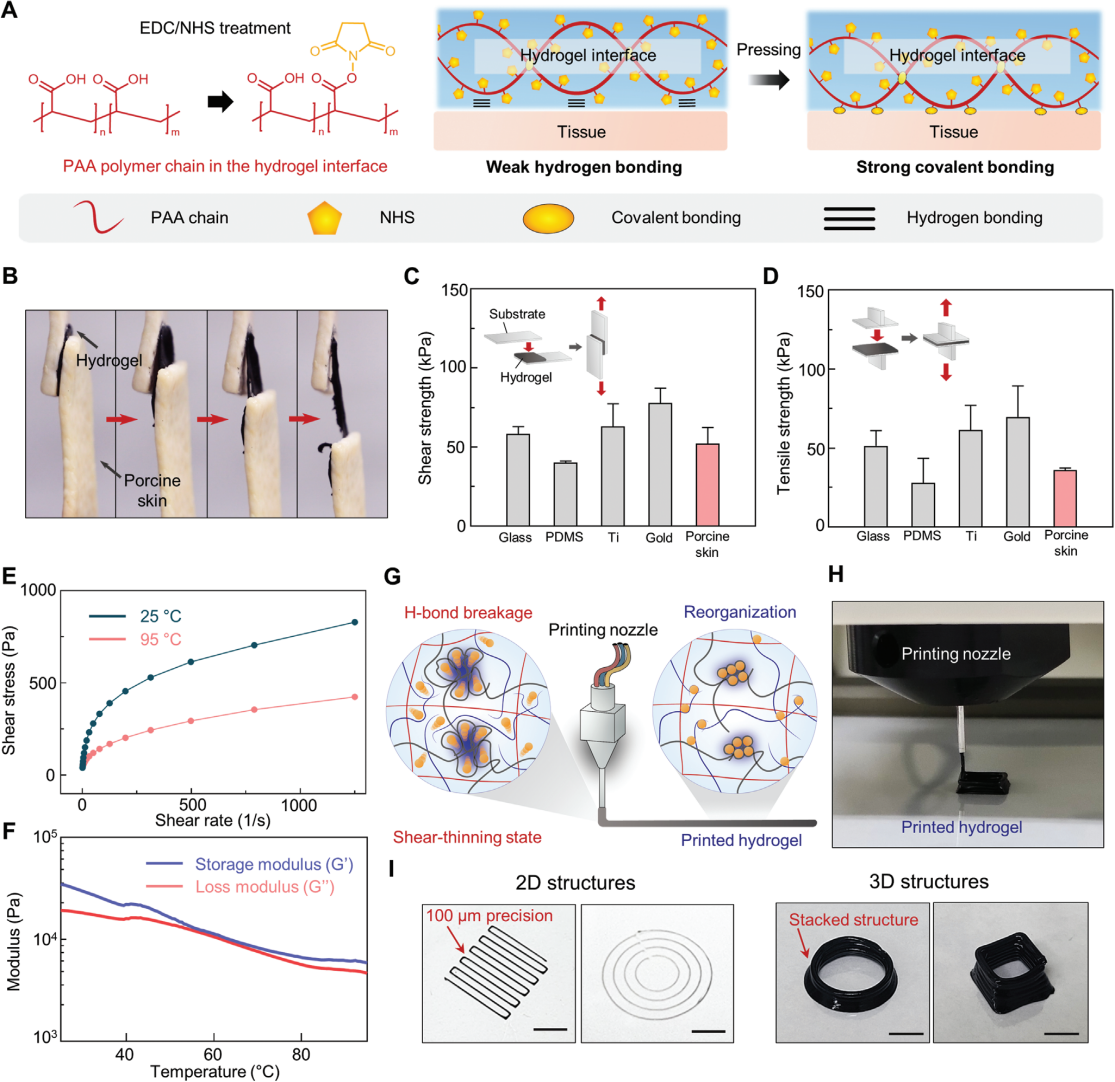
Adhesive and 3D‐printable properties of the hydrogel. A) Schematic illustration of the tissue‐adhesive property of the hydrogel treated with NHS. B) Sequential photographs of lap shear test of the hydrogel adhered between two porcine skins. C,D) Shear and tensile strengths of the hydrogel against various substrates and porcine skin (n = 3: n is the sample size for each group). E,F) Rheological studies of the hydrogel including shear stress as a function of shear rate and moduli as a function of temperature. G,H) Schematic illustration and photographic image of the thermal‐assisted 3D‐printed hydrogel. I) Various 2D geometries and 3D structures of the printed hydrogel. (Scale bar: 2 mm for 2D structures and 4 mm for 3D structures)

Bioelectronics should be able to electrically measure from and stimulate the tissue surfaces for diagnostic and therapeutic applications. Accordingly, conformal contact and adhesion between the bioelectronics and tissue surface are required for efficient electrical‐signal transmission.^[^ ^43^ ^]^ Frequent movements of the human body can detach the adhered bioelectronics from the target tissue, and even their slight displacement can lead to poor signal resolution. Thus, stable interfacing between the tissues and the hydrogel is required to further prolong the bioelectronic functionalities of the hydrogel. In addition, as the bioelectronic devices are an integration of various materials, the hydrogel should also be able to adhere to other bioelectronic materials to enable the designing of complex bioelectronics systems. An additional chemical treatment of the hydrogel endows it with a strong adhesive property with respect to tissues and other materials. After functionalizing carboxyl groups in PAA with N‐hydroxysuccinimide ester (NHS), the hydrogel can covalently bond with surfaces having primary amine groups (**Figure** **5**A).^[^ ^44^ ^]^ Upon contact between the hydrogel and the primary amine‐functionalized surfaces, the two surfaces weakly bond via H‐bond and after approximately 5 min, the two surfaces strongly bond through NHS carbodiimide chemistry. Because soft tissues have abundant primary groups (e.g., in lysine), fCNT/TA/PVA/PAA‐NHS can strongly adhere to tissues.^[^ ^45^ ^]^ In addition, the fCNT/TA/PVA/PAA‐NHS can adhere to various electronic‐device materials functionalized with primary amine groups. Figure S13, Supporting Information, shows schematics of primary‐amine‐functionalized device materials. Thus, the hydrogel can be integrated with other electronic materials to design various bioelectronic systems. We performed a lap shear test and tensile test to examine the interface adhesion force of the hydrogel. Figure 5B shows an optical image of the hydrogel sandwiched between two porcine skin substrates and under the lap shear test. The image clearly shows a distinct adhesive property as the hydrogel can endure shear movements. Figure 5C,D and Figure S14, Supporting Information, show the quantification of interfacial adhesiveness through the lap shear and tensile tests. The hydrogel exhibited high shear and tensile adhesion strength to various surfaces in the range of 40–75 kPa. As the internal organs are far more hydrated, the wet adhesion ability is an important issue to be addressed for in vivo bioelectronic applications. Yet, achieving reliable wet adhesion is challenging as the adhesion can be hampered by the interfacial water molecules, and we noticed that the nonswellable nature of our hydrogel makes it hard to remove the interfacial waters. By manually removing excessive water on organ surfaces by using gauze, the hydrogel can adhere to internal organ surfaces (Figure S15, Supporting Information). The results suggest that the hydrogel can stably interface with soft tissues or can be integrated with other materials to implement sophisticated bioelectronic devices. Furthermore, we noted that the hydrogel could be 3D‐printed. 3D printability allows the hydrogel to be integrated into the complex and versatile form of bioelectronic devices with sufficient spatial resolution. To date, fabrication methods for processing bulk‐form hydrogels into precise patterns and shapes are lacking. In addition, high water contents render them incompatible with fabrication technologies involving high temperatures, such as thermal drawing.^[^ ^6^ ^]^ Therefore, the printability of our hydrogel implies a huge impact on the commercialization of hydrogel bioelectronics. We conducted rheological tests to examine the printable property of the hydrogel. Figure 5E shows shear stress as a function of shear rate, which indicates the pseudoplastic fluid behavior of the hydrogel capable of shear thinning. Under heat treatment at 95 °C, shear stress decreased owing to H‐bond breakages. G′ and G″ were measured as a function of temperature to further investigate the thermoresponsive property of the hydrogel (Figure 5F). A gradual decrease in moduli was observed with increasing temperature; however, the storage modulus was higher than the loss modulus in all temperature regions. The rheological analyses suggest that heat induces a greater shear‐thinning tendency of the hydrogel while still retaining its elastic nature rather than transitioning to a liquid state. Thus, the hydrogel does not laterally spread after thermal‐assisted printing; rather, it restores cleaved H‐bonds to reorganize the initial network (Figure 5G,H). We demonstrated that the hydrogel could be printed in various geometries with 100‐µm precision and also as stacked 3D structures (Figure 5I,J). Further treatments such as chemical and thermal treatment could render the hydrogel adhesive and printable, respectively. Combining its adhesiveness with diverse electronic materials and printability with micron‐level precision would enable the hydrogel to be integrated within complex bioelectronic systems as a sophisticated device.

### 2.5 Bioelectronic Applications

**Figure 6:**
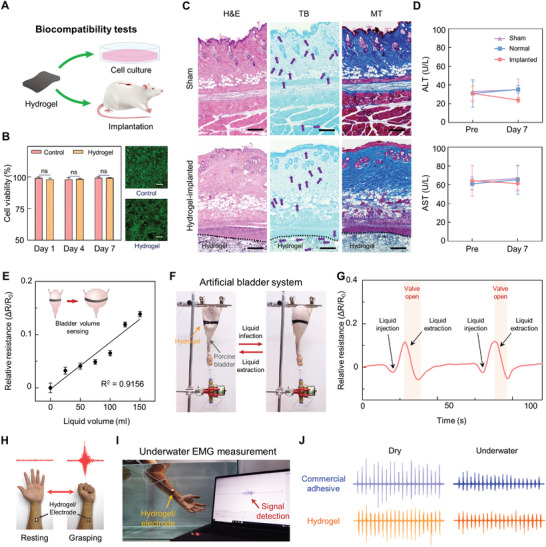
Biocompatibility and bioelectronic applications of the hydrogel. A) In vitro and In vivo biocompatibility of the hydrogel. B) Statistical analysis of in vitro biocompatibility test of the hydrogel (ns: no significant differences; n = 4: n is the sample size for each group) and the representative fluorescent images after 7 days (scale bar: 50 µm). The statistical analysis was conducted with an unpaired t‐test (*P < 0.05, **P < 0.01, ***P < 0.001, and ****P < 0.0001; ns: no significant difference). C) Representative histological images stained with hematoxylin and eosin (H&E), Masson's trichrome (MT), and Toluene blue (TB) for assessment of the biocompatibility of the hydrogel in vivo after 3 days of subcutaneous implantation (scale bars = 50 µm). Purple arrows indicate mast cells. D) Serum levels of Aspartate aminotransferase (AST) and alanine aminotransferase (ALT) before (Pre) and at 7 days after the implantation of the hydrogel (n = 6 for the implanted group and n = 3 for the sham and normal groups: n is the sample size for each group). The statistical analysis was conducted by using an unpaired t‐test. E) Relative resistances of the hydrogel attached to the ex vivo porcine bladder as a function of injected liquid volume within the bladder (n = 10: n is the sample size for each volume point). F) Photograph of an artificial bladder system capable of injecting and extracting liquids. G) Real‐time resistive responses of the hydrogel on the porcine bladder in which liquid injection and extraction are controlled. H) An optical image of the hydrogel detecting the EMG signal from a grasping human hand. I) Photograph of underwater EMG signal detection. J) Measured EMG signals with commercial adhesive and the developed hydrogel under dry conditions and underwater.

Bioelectronics record various biosignals from the human body for medical diagnostics. As daily healthcare systems attract increasing demand, real‐time and long‐term functioning of bioelectronics under daily activities should be provided. To realize real‐time and long‐term recording with clear biosignals, the bioelectronics should robustly interface with tissues. In particular, the bioelectronics should strongly attach to the tissue and endure dynamic conditions. Additionally, they must be conformally attached to the target tissue for accurate biosignal detection. Because of its multifunctionality, including swelling resistance, softness, mechanical durability, electrical performance, and strong adhesiveness, the hydrogel can afford diverse practical bioelectronic applications. We demonstrated the applicability of the hydrogel to organ strain sensing and electrophysiological signal detection. First, we performed the in vitro and in vivo biocompatibility tests of the hydrogel to verify its nontoxicity for human‐body applications (**Figure** **6**A and Figure S16, Supporting Information). The hydrogel was incubated with NIH 3T3 fibroblasts in a cell culture media for 7 days, and a live/dead assay was conducted (Figure 6B). The result showed that no significant differences in cell viability existed between fibroblasts with the hydrogel and without it for 7 days. We further implanted the hydrogel into mouse subcutaneous tissues and conducted histological and hepatotoxicity assessments. The hydrogel‐implanted group was compared with a normal group of intact mice and a sham group that was surgically incised. The adjacent skin tissues were stained with hematoxylin and eosin (H&E), Masson's trichrome (MT), and Toluidine blue (TB) after 3 and 7 d (Figure 6C and Figure S17, Supporting Information). The histological stainings revealed that the hydrogels were not degraded within subcutaneous space for 7 d and no significant inflammatory responses were observed in surrounding tissues at both 3 and 7d. TB stains an immune cell of connective tissue, mast cell, and it reveals a degree of inflammatory responses. We identified comparable numbers of mast cells between the sham and hydrogel‐implanted groups. Serum levels of alanine aminotransferase (ALT) and aminotransferase (AST) were measured before and after 7 d of implantations to evaluate the hematotoxicity (Figure 6D). There were no statistical significances in serum levels of ALT and AST between normal, sham, and hydrogel‐implanted groups at each time point, suggesting that the implantation of the hydrogel did not exert hematological inflammation. The biocompatibility tests indicate that the hydrogel is compatible with biological tissues and can thus be applied as a bioelectronic device. Numerous patients with neurogenic lower‐urinary‐tract dysfunction suffer from reduced bladder sensation and low quality of life.^[^ ^46^ ^]^ To deal with this issue, stable continuous monitoring of the bladder volume is critical issue. Accordingly, we selected a bladder volume detection application to verify the strain‐sensing property of the hydrogel. We utilized and ex vivo porcine bladder to design an artificial bladder system. A solenoid valve connected with a custom‐made control circuit was used to control the liquid injection into and extraction from the bladder outlet. Each resistive change on every 25‐mL liquid injection to the bladder was recorded, and their linear relationship with *R* ^2^ = 0.9156 was confirmed (Figure 6E). Because the hydrogel exhibited a repeatable resistive response to multiple strains, it could stably exhibit long‐term monitoring of the bladder volume. To demonstrate real‐time bladder volume detection, the artificial bladder system was controlled to inject and extract liquid (Figure 6F). Movie S1, Supporting Information, and Figure 6G show the resistive response of the hydrogel against the injection and extraction of liquids. The immediate resistive changes corresponded to the volume of liquids as the hydrogel could conformally interface with the bladder. The results indicate the reliable organ strain‐sensing ability of the hydrogel. Next, we tested the applicability of the hydrogel to EMG detection. EMG signals provide electrical activity in muscle activity, which can be used to diagnose neuromuscular abnormalities and assess sports activity. In particular, underwater EMG sensing is important in rehabilitation and sports medicine applications; thus, it should be addressed.^[^ ^47^ ^]^ We first confirmed the EMG‐sensing ability of our hydrogel by adhering the hydrogel to the human arm. The grasping of the hand was detected as a clear signal (Figure 6H). Notably, our hydrogel could provide reliable underwater EMG signal monitoring owing to its remarkable adhesiveness and nonswellability. Therefore, we also tested its EMG‐sensing ability in a water tank (Figure 6I and Movie S2, Supporting Information). Figure 6J shows the monitored EMG signals using commercial adhesives and the hydrogel under both dry and underwater conditions. The hydrogel revealed a stable EMG‐recording ability comparable to a commercial adhesive that is widely adopted in the industry. The hydrogel was demonstrated to serve as a stable EMG sensor even under water and in dynamic situations. Through the application of the hydrogel to representative and high‐demand bioelectronics, we verified its applicability to various challenging bioelectronics.

## 3 Conclusion

We developed a multifunctional hydrogel for implementing long‐term functioning bioelectronics. Although there have been reported various TA‐based hydrogels, achieving all the properties such as softness, self‐healing ability, toughness, and conductivity involved a trade‐off and was challenging. Our hydrogel overcomes several conventional trade‐offs of hydrogels owing to its dynamic nanoconfinement network. The nanoconfined region effectively dissipates fracture energies and the nonconfined regions ensure dynamic networks for softness and rapid self‐healing ability. Therefore, the hydrogel has low Young's moduli similar to that of soft tissues while exhibiting high toughness to endure externally applied stresses. Even if the hydrogel is damaged, it can rapidly and perfectly self‐heal within 5 min. Notably, the hydrogel is nonswellable; therefore, multifunctionality can be achieved in wet tissue environments in the long term. The dynamic network also promotes electrical robustness by reconstructing the conducting pathway of the hydrogel. Further treatments of the hydrogel enable the strengthening of tissue interfacing via tissue adhesiveness of the hydrogel and printing in versatile forms for realizing various bioelectronic device designs. We envision that our hydrogel‐based bioelectronics could be practically utilized in daily healthcare systems because the hydrogel with long‐term durability can protect its bioelectronic functionalities against dynamic and harsh conditions occurred during daily usage. In a future study, we will further explore the diverse aspects of hydrogel. For example, the hydrogel revealed viscoelasticity with fast relaxation time. Exploring the diverse cellular responses to the viscoelasticity of materials is currently a critical issue.^[^ ^29^ ^]^ By modulating the viscoelasticity of the hydrogel, it could be further developed into a therapeutic implant. In addition, its electrochemical properties will be thoroughly investigated to utilize it as an electrochemical biosensor for detecting various biomolecules. Hydrogel holds excellent potential for studies and application in the biomedical field. Such future studies will broaden the applicability of hydrogel in bioelectronics and rationally bridge biology and electronics.

## 4 Experimental Section

### Synthesis of fCNT

fCNT was synthesized via the oxidation of multiwalled CNTs (MWCNTs) using KMnO_4_ as an oxidizing agent. Four grams of MWCNTs (JenoTube 20A, JEIO, South Korea, diameter of 15–25 nm, length of 20–100 µm, and thickness of 7–12 layers) were mixed with KMnO_4_ in 200 mL of sulfuric acid. While maintaining the temperature at 35 °C, the mixture was stirred at 450 rpm for 1 h. After the addition of 350 mL of deionized (DI) water to the mixture, 80 mL of H_2_O_2_ was added sequentially. To eliminate the remaining reactants and acidic solvents, the fCNT solution was washed by filtering the reacted mixture with DI water on cellulose filter paper.

### Preparation of fCNT/TA/PVA/PAA Hydrogel

To prepare the hydrogel, 20%, 30%, and 40% w/v TA (Sigma‐Aldrich) was initially mixed into PVA solution (20% w/v in DI water, Mw 89 000–98 000) and stirred for 2 h by heating at 90 °C to obtain a TA/PVA gel. The 1% w/v fCNT was further mixed into the TA/PVA gel followed by 2‐h stirring and heating at 90 °C. The resulting fCNT/TA/PVA gel was freezed at −20 °C for 8 h and thawed at room temperature for 3 h to prepare the fCNT/TA/PVA hydrogel. To introduce the PAA chain into the fCNT/TA/PVA hydrogel, the fCNT/TA/PVA hydrogel was dried at 37 °C for 1 h and further annealed at 100 °C for 1 h. The dried thin fCNT/TA/PVA film was soaked in an acrylic acid solution (30% w/w acrylic acid, 0.03% w/w N,N′‐bis(acryloyl)cystamine, and 0.15% w/w 2,2′‐azobis(2‐methylpropionamidine) dihydrochloride in deionized water) for 2 h. The immersed film was heated at 70 °C for 30 min to form the fCNT/TA/PVA/PAA hydrogel.

To enable the hydrogel adhesive property, NHS ester groups were introduced into the PAA chain. The fCNT/TA/PVA/PAA hydrogel was soaked in a 2‐(N‐morpholino)ethanesulfonic acid (MES) buffer containing 1‐ethyl‐3‐(‐3‐dimethylaminopropyl) carboiimide (0.5% w/w) and NHS sodium salt (0.25% w/w) for 5 min at room temperature.

### Characterizations

The chemical structures of hydrogels were analyzed through FT‐IR (Vertex 70, Bruker, USA) and XPS (K‐alpha, Thermo, UK). The FT‐IR spectra were acquired with a spectral range of 4000 to 350 cm^−1^. XPS was performed on a circular sampling area of freeze‐dried samples having a diameter of 400 µm using an XPS spectrometer equipped with an X‐ray source of Al K‐*α* line. The crystallographic structures of the hydrogels were characterized via X‐ray diffraction (SmartLab, Rigaku, Japan). To demonstrate the nanoconfinement structures of the fCNT/TA/PVA/PAA hydrogel, AFM (NX‐10, Park Systems, Korea) and TEM (JEM‐2100Plus, JEOL, Japan) analysis were performed. The AFM images were obtained from freeze‐dried hydrogels by using the phase image mode of AFM. To obtain TEM images, the hydrogels were initially freeze‐dried and ultrathin‐sectioned with a thickness of ≈100 nm using an ultramicrotome (PowerTome PC, RMC, USA). Then, TEM was performed using an electron microscope at a 200‐kV acceleration voltage.

### Mechanical Characterizations

All the tensile tests of the hydrogels were conducted on a mechanical testing machine (MultiTest 2.5‐DV, Mecmesin, UK) with a 50‐N load cell at a speed of 50 mm min^−1^. The sample size was approximately 10 × 10 × 1 mm^3^. The cyclic tensile–compression test was performed by using the same equipment and at a speed of 10 mm min^−1^. The rheological properties of the hydrogel were analyzed using a rheometer (MCR102, Anton Paar, Austria). The hydrogel samples were loaded between 25‐mm‐diameter parallel plates with ≈1‐mm distance. A frequency sweep test was performed at a frequency range of 0.1–100 rad s^−1^ at a constant 1% strain, and a thermal analysis was conducted by varying the temperature from 25 to 95 °C at a constant frequency of 50 rad s^−1^ and 1% strain.

### Electrical Characterization

Electrical conductivity (*σ*) was measured using the modified four‐point probe method. By applying an AC voltage (±1 V, 1000 Hz), voltage (*V*) and current (*I*) were recorded using a probe station (MST4000A, MSTECH, Korea). The thickness (*T*), length (*L*), and width (*W*) were measured using an optical microscope (SMZ645, Nikon, Japan). The electrical conductivity (*σ*) was calculated using the equation: *σ* = (*L* × *I*)/(*W* × *T* × *V*).

The electrical impedance was characterized by using a potentiostat (SP‐200, Bio‐Logic, USA). The hydrogel samples were loaded between two platinum electrodes with a 10 mm × 10 mm area and ≈200‐µm thickness. The two electrodes were connected to the potentiostat, and EIS measurement was conducted. To characterize the electrical resistance of the hydrogel, the output voltage and current across the hydrogel were measured in real‐time using an electrometer (Keithley 6514, Tektronix, USA).

### Surface Functionalization of Device Materials

To implement strong adhesion between the hydrogel and various device material surfaces through covalent bonds, the device material surfaces were functionalized with primary amine groups (Figure S5, Supporting Information). For the functionalization of glass, PDMS, and Ti, the substrates were initially treated with oxygen plasma (70‐W power, 2 min) using a plasma system (COVANCE, Femto Science, Korea) to hydroxylate the surfaces. Next, silanization through APTES was carried out according to a previously reported method.^[^ ^39^ ^]^ Gold was functionalized with cysteamine hydrochloride solution (50 mm in DI water) according to the previously reported method.^[^ ^39^ ^]^ The amine‐functionalized substrates were finally washed with isopropyl alcohol and dried under nitrogen gas flow.

### Adhesion Force Tests

The adhesion force of the hydrogel to various substrate surfaces was assessed by conducting lap shear and tensile tests. Both tests were performed using a mechanical testing machine (MultiTest 2.5‐DV, Mecmesin, UK) equipped with a 2500‐N load cell. For both tests, the amine‐functionalized device materials and porcine skin substrates with dimensions of 76 × 26 × 1 mm^3^ were prepared for the tests, and the hydrogel was placed between the two substrates with an adhesion area of 10 × 10 mm^2^. Then, the test was conducted at a speed of 10 mm min^−1^. From the result, the adhesion strengths were calculated as the maximum force divided by the adhered area in kilopascals.

### In Vitro Biocompatibility Test

The hydrogel was prepared with dimensions of 10 × 10 × 1 mm^3^. Afterwards, the NIH‐3T3 fibroblast cells (0.5 × 10^5^ cells per mL) were directly cultured with the prepared hydrogel in 1 mL of DMEM supplemented with 10% bovine calf serum and 1% penicillin–streptomycin. A viability test was conducted using a Live/Dead kit (L3224, Invitrogen, USA) according to the manufacturer's instructions. To visualize the viability of the cells, a laser scanning confocal microscope (LSM 700, Carl Zeiss, Germany) with a 10× magnification was used.

### In Vivo Biocompatibility Test

In vivo biocompatibility of the hydrogel was tested by subcutaneously implanting (5 × 5 × 5 mm^3^) into mice for 3 and 7 days. The animal procedures were approved by the Institutional Animal Care and Use Committee of Yonsei University College of Medicine (permit number: 2022‐0321). All animal experiments were conducted at a facility accredited by the Association for the Assessment and Accreditation of Laboratory Animal Care (AAALAC). In this facility, animals were housed with food and water given ad libitum under alternating 12‐h light/dark cycles, according to animal protection regulations. The scaffolds were subcutaneously implanted in 6‐week old male CD‐1 (ICR) mice (Orient Bio Incorporation, Seongnam, Korea) that had been anesthetized with ketamine (100 mg kg^−1^), and Rompum (10 mg kg^−1^). To test the hepatotoxicity of the hydrogel, serum was taken through retro‐orbital bleeding before and after 7 days of implantation (n = 3 for Normal and Sham group, n = 5 for hydrogel‐implanted group). The serum levels of AST and ALT were measured using a biochemical analyzer (Dri‐Chem 4000i, Fujifilm, Japan). Histological analysis was performed at 3 and 7 days of the post‐implantation (n = 3 for Normal, and n = 3 for hydrogel‐implanted group). The mice were euthanized, and the implants were retrieved along with the surrounding skin tissues and fixed in 10% formalin (Sigma‐Aldrich). The fixed tissues were embedded in paraffin blocks. Sections of each sample at 4 µm thickness were mounted onto slides for histological staining with Hematoxylin and Eosin (H&E), Toluidine blue (TB), and Masson's Trichrome (MT).

### Bioelectronic Applications

An artificial bladder system was designed with a solenoid valve with a customized control circuit. The injection and extraction of DI water were controlled by the circuit. An electrometer (Keithley 6514, Tektronix, USA) was used to read the resistances of the hydrogel.

A commercial kit (BITalino (r)evolution board kit BLE, PLUX Biosignals, Portugal) was used to detect EMG signals. A three‐lead system was used and connected the developed hydrogels to each lead. Two hydrogels were attached to a forearm and the other to a bicep as a reference. A commercial adhesive (Red Dot, 3M, US) was used for comparison with the hydrogel. The grasping action of the hand was detected with EMG signals from the hydrogel and the commercial adhesive. The differences between groups were tested by unpaired t‐tests.

### Informed Consent

The human research participants are shown in the photographs in Figure 6H, I, and Figure S9, Supporting Information, consent to the publication of these images. We confirmed that obtaining institutional review board approval is not required for conducting research using wearable devices simply contacting human skin without physical modification or invasive measurement.

### Statistical Analysis

All experiments were performed with a sample size of at least N = 3 for each data point. GraphPad Prism 8 software (Graphpad Software Inc., USA) was utilized to conduct statistical analyses for all experiments except in vivo studies. The significant differences between groups were compared by using unpaired t‐tests. Statistical analyses for in vivo studies were performed using SPSS software (IBM, Armonk, NY, version 26.0).

## Conflict of Interest

The authors declare no conflict of interest.

## Supporting information

Supporting Information

Supporting Video 1

Supporting Video 2

## Data Availability

The data that support the findings of this study are available from the corresponding author upon reasonable request.
