# Supplementary material for: Intrinsically Nonswellable Multifunctional Hydrogel with Dynamic Nanoconfinement Networks for Robust Tissue‐Adaptable Bioelectronics
Source: Adv Sci (Weinh). 2023 Feb 17;10(12):2207237. doi: 10.1002/advs.202207237 (PMC10131858; doi:10.1002/advs.202207237)
Supplement: Supplementary file 1 — Supporting Information [file ADVS-10-2207237-s002.pdf]

ADVANCED SCIENCE
Open Access

Supporting Information

for *Adv. Sci.*, DOI 10.1002/advs.202207237

Intrinsically Nonswellable Multifunctional Hydrogel with Dynamic Nanoconfinement Networks for Robust Tissue-Adaptable Bioelectronics

*Jae Park, Ju Yeon Kim, Jeong Hyun Heo, Yeonju Kim, Soo A Kim, Kijun Park, Yeontaek Lee, Yoonhee Jin, Su Ryon Shin, Dae Woo Kim and Jungmok Seo**

Supporting Information

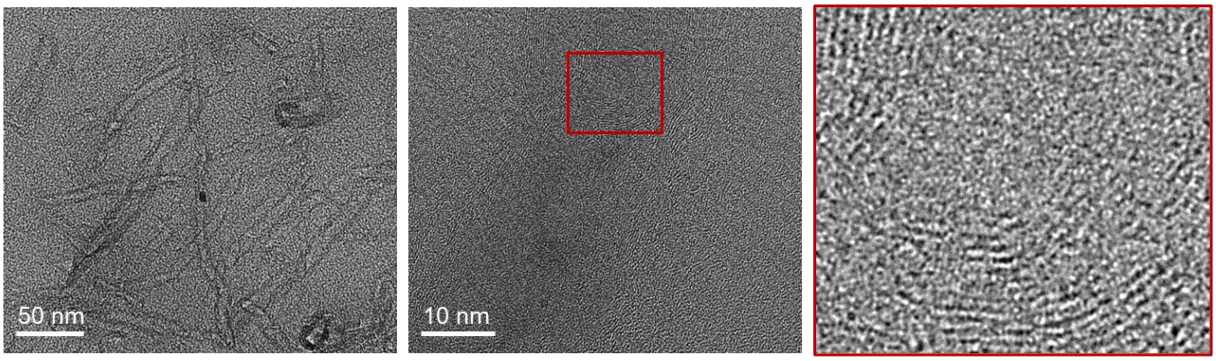

Figure S1. TEM images of fCNT/TA/PVA/PAA.

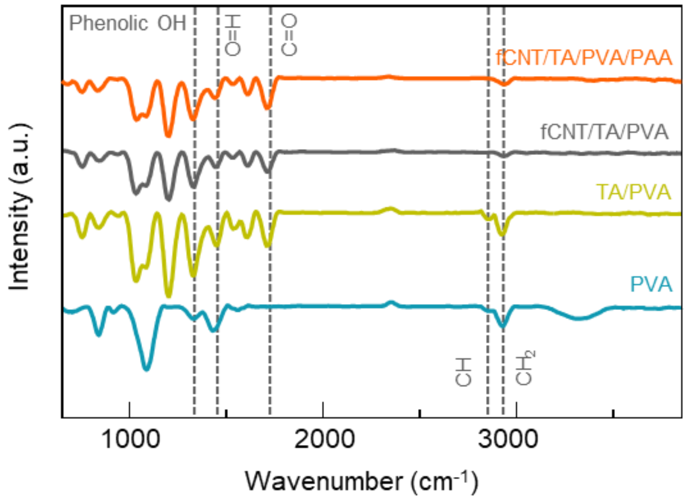

Figure S2. FT-IR spectra of hydrogels for each fabrication step.

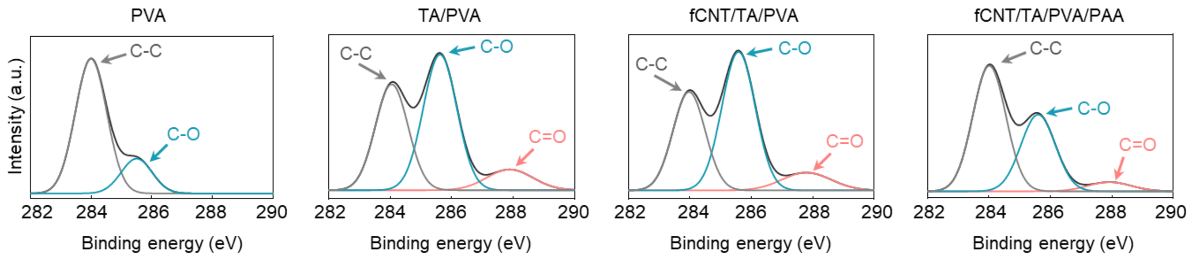

Figure S3. XPS characterization of hydrogels at each fabrication step.

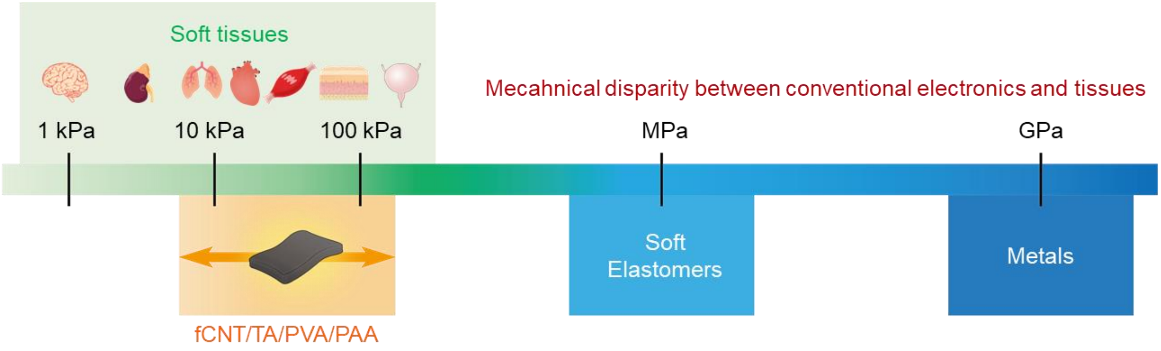

Figure S4. Young’s moduli of various soft tissues, conventional electronic materials, and the developed hydrogel (fCNT/TA/PVA/PAA).

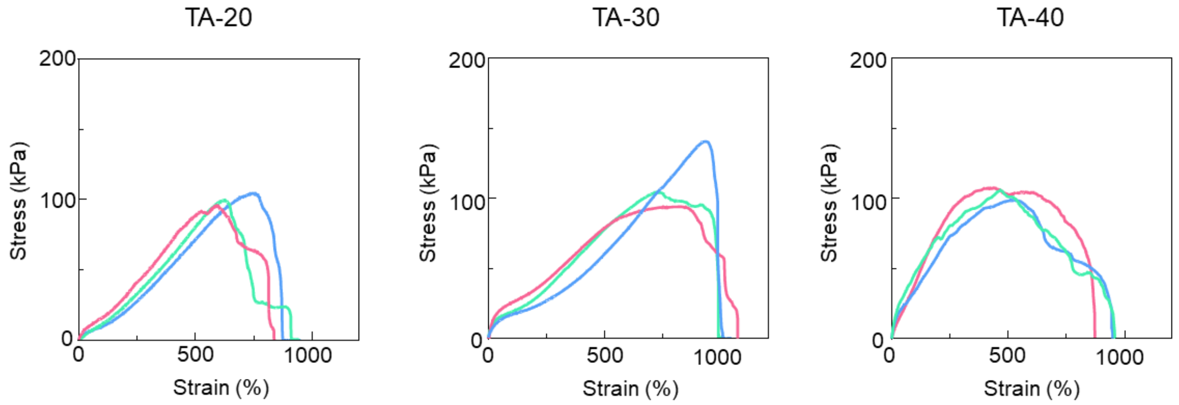

Figure S5. Tensile tests for the hydrogels with varying TA concentrations.

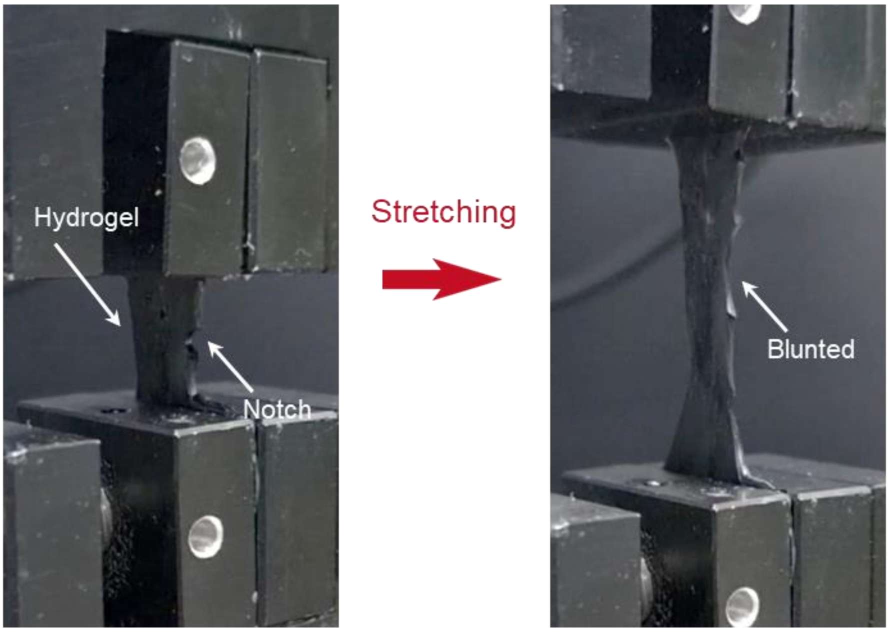

Figure S6. Optical image of the hydrogel showing notch-insensitive property.

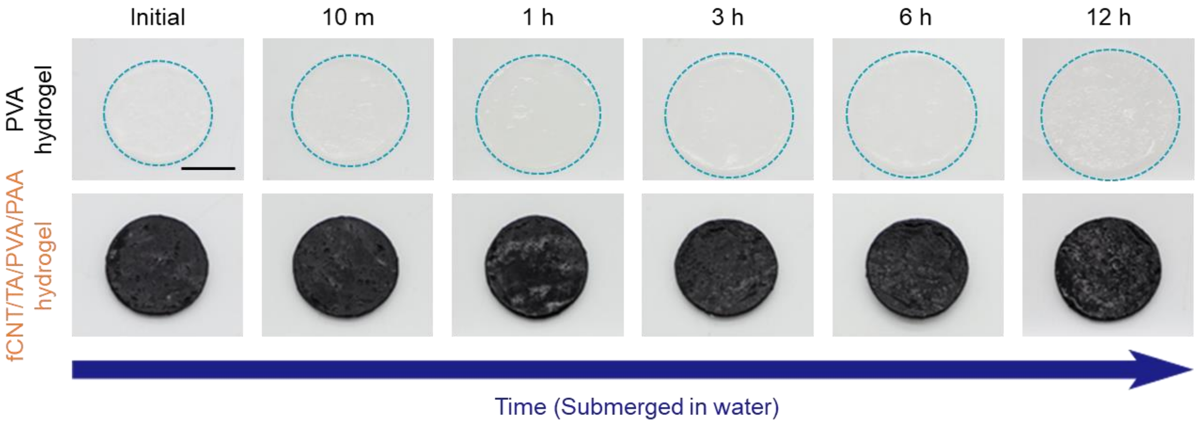

Figure S7. Photographs of the swelling behavior of PVA and fCNT/TA/PVA/PAA hydrogel submerged in water for 12 h. (Scale bar: 5 mm)

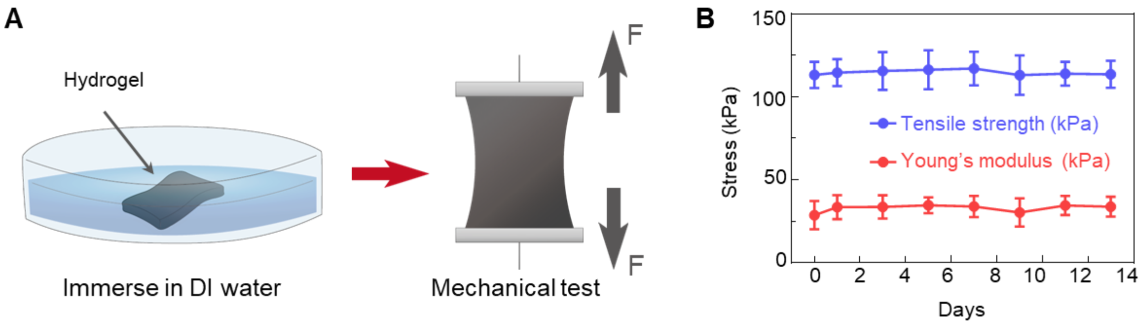

Figure S8. Mechanical tests of the hydrogel immersed in water for 13 days. A) Schematic illustration of the experimental method. The hydrogel was immersed in water and then its tensile test was conducted. B) Tensile strength and Young' modulus of the hydrogel as a function of immersion time (n = 3: n is the sample size for each time point).

A B

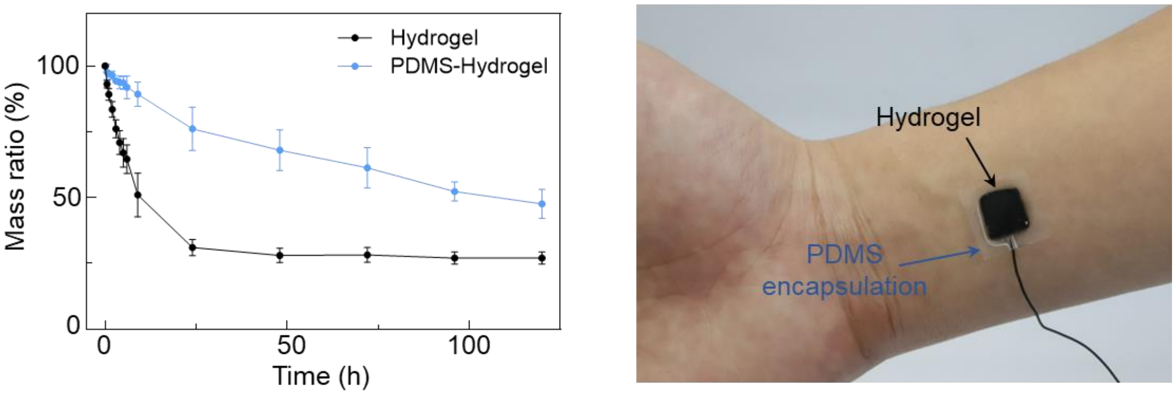

Figure S9. The PDMS-encapsulated hydrogel for preventing the hydrogel's water loss. A) Variations in the masses of the bare hydrogel and encapsulated hydrogel as a function of time (n = 3: n is the sample size for each time point). B) An optical photograph showing the PDMS-encapsulated hydrogel as a wearable patch.

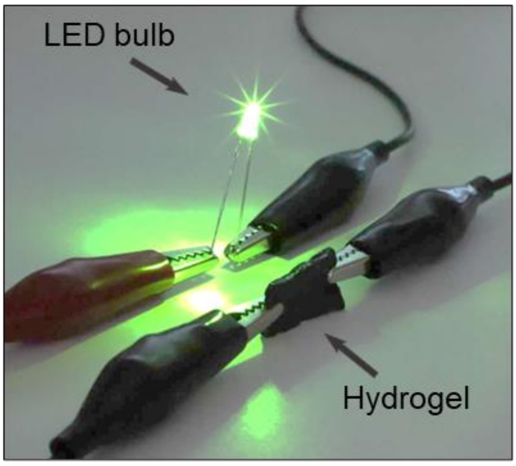

Figure S10. Optical image of the hydrogel connected to an LED.

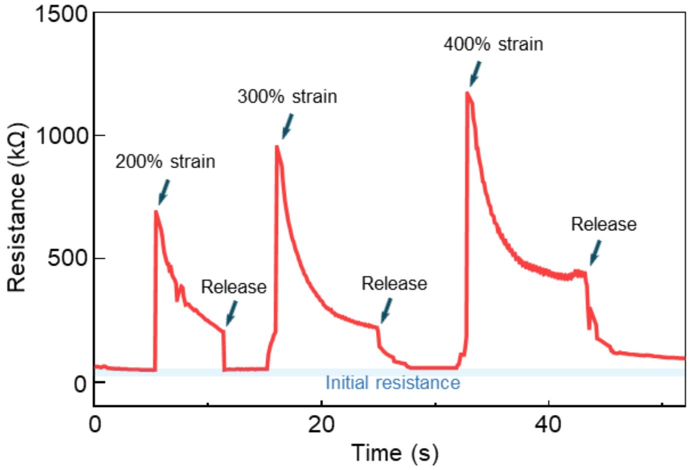

Figure S11. A real-time resistance measurement for the hydrogel upon sequential strain applications of 200, 300, and 400%.

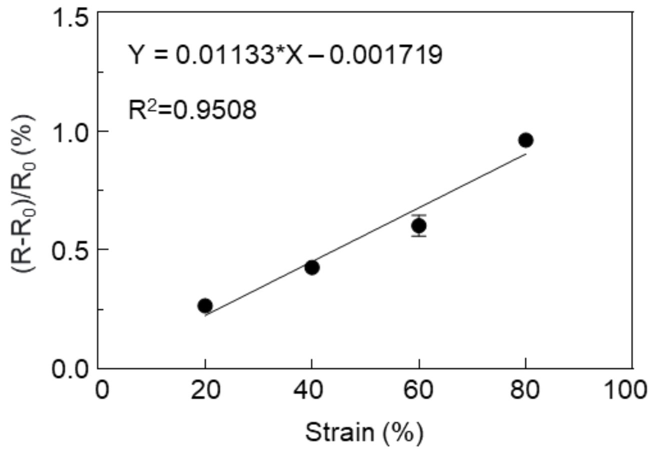

Figure S12. Relative resistances of the hydrogel as a function of applied strains and their linear fitting (n = 4: n is the sample size for each strain point).

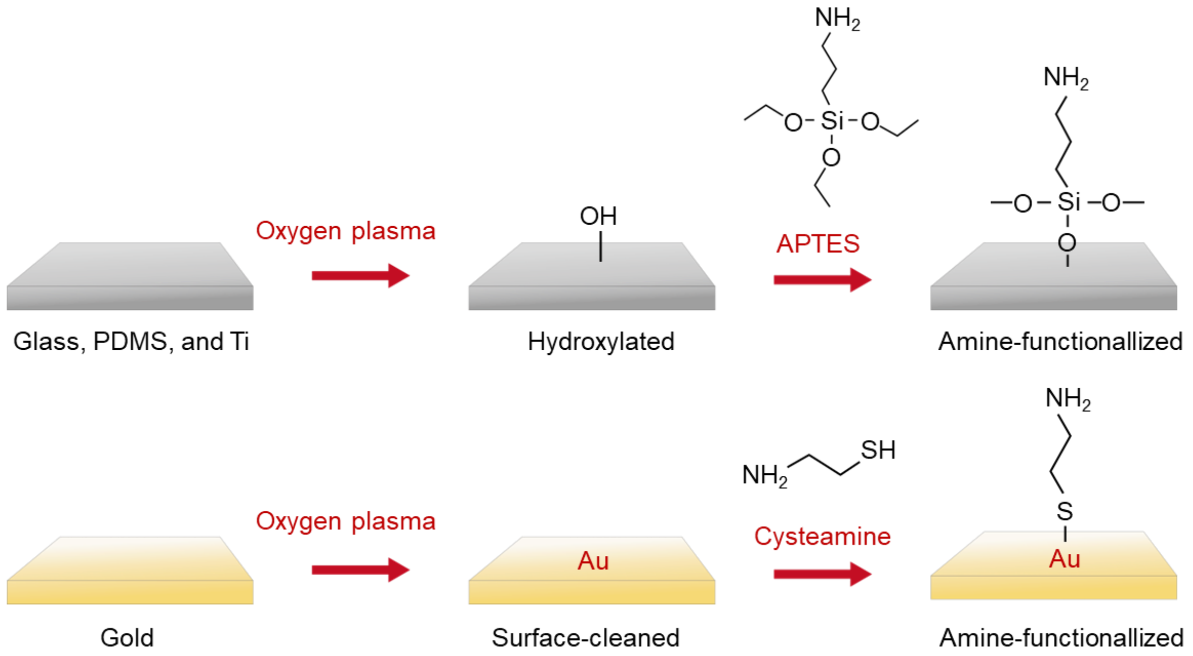

Figure S13. Schematic illustration of the process of primary amine group functionalization of various substrates.

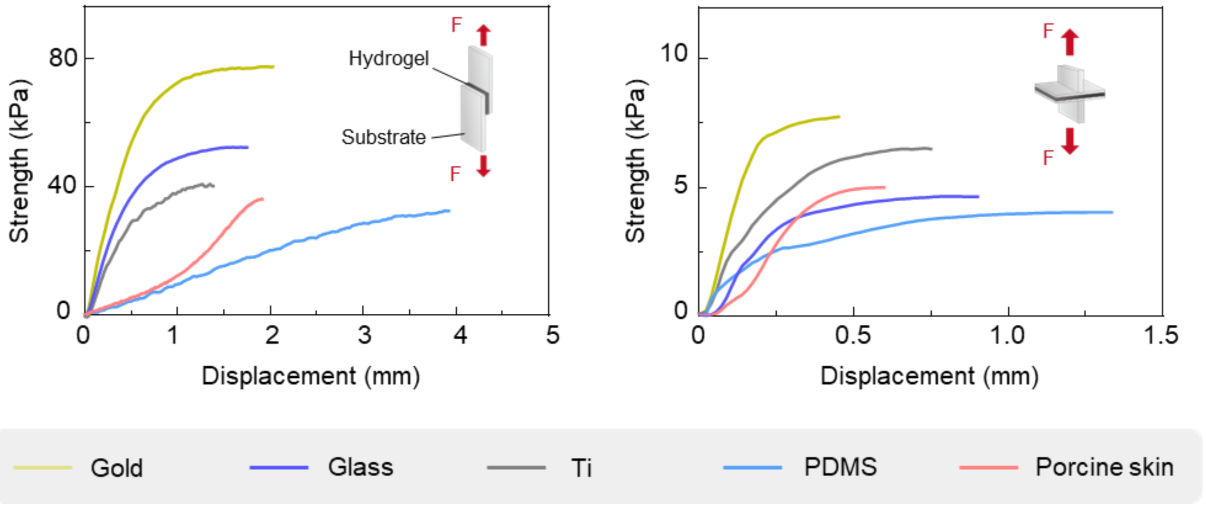

Figure S14. Representative curves for shear and tensile adhesion tests of the hydrogel against various substrates.

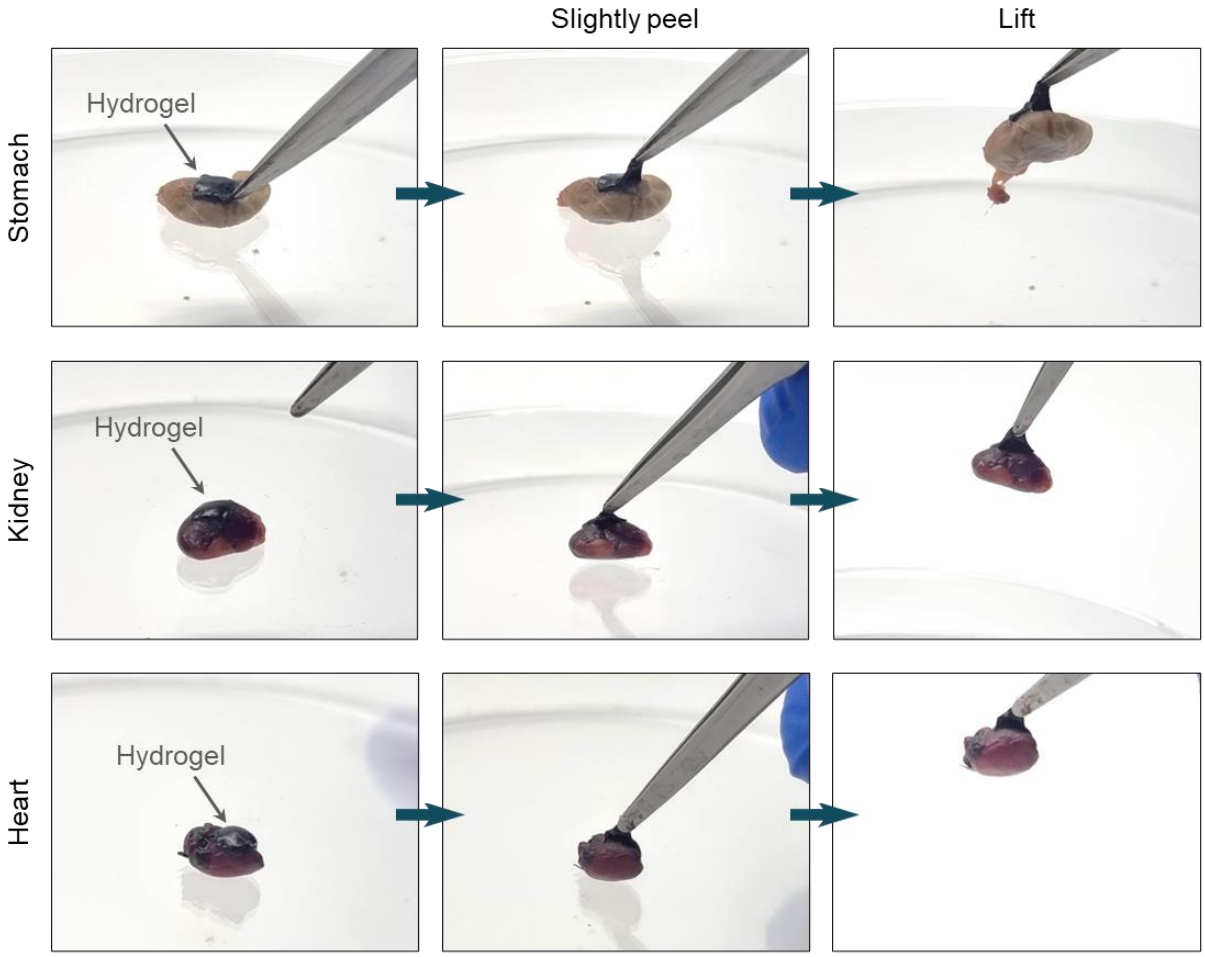

Figure S15. Photographic image showing the hydrogel adhered to various *ex vivo* mouse organs after physically removing excessive waters (stomach, kidney, and heart).

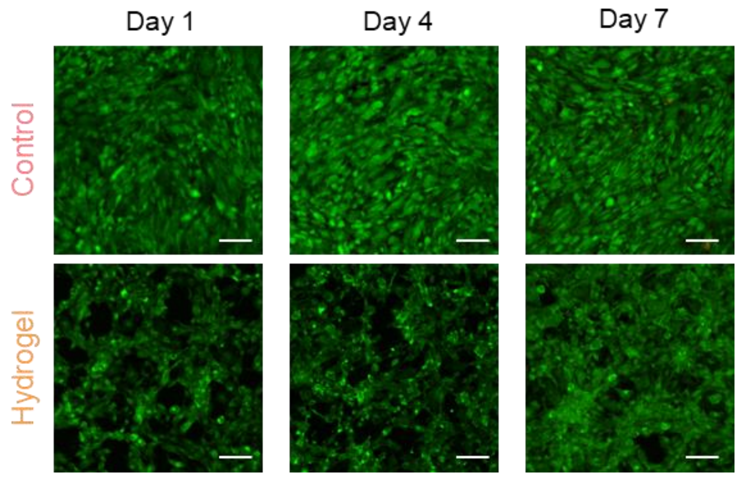

Figure S16. In vitro biocompatibility of the hydrogel in a live/dead assay of fibroblast cell line (NIH 3T3).

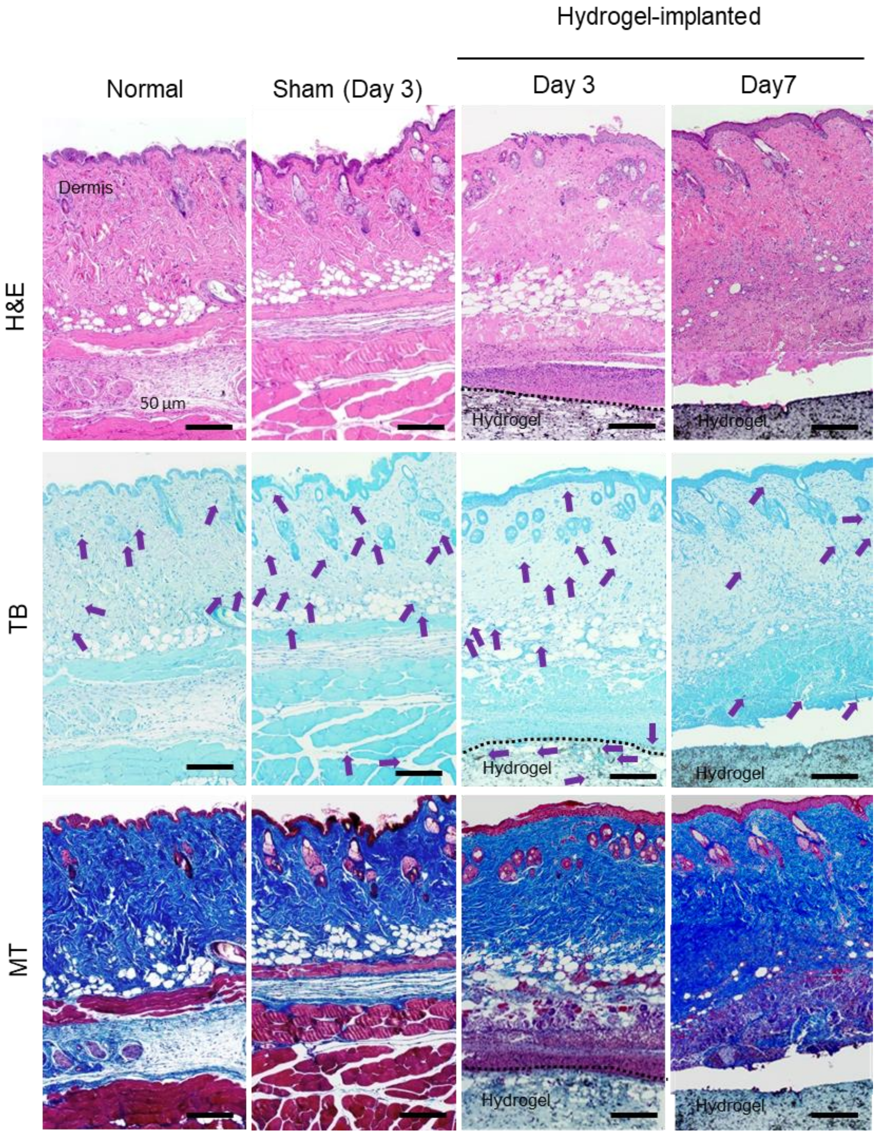

Figure S17. Histological images of the normal, sham, and hydrogel-implanted tissues stained with hematoxylin and eosin (H&E), Toluidine blue (TB), and Masson's trichrome (MT) for in vivo biocompatibility test (scale bars = 50 μm). The images for the hydrogel-implanted group include tissue stained after seven days. Purple arrows indicate mast cells.
